# An effective ex-vivo approach for inducing endothelial progenitor cells from umbilical cord blood CD34^+^ cells

**DOI:** 10.1186/s13287-017-0482-9

**Published:** 2017-02-07

**Authors:** Meng Qin, Xin Guan, Huihui Wang, Yu Zhang, Bin Shen, Qingyu Zhang, Wei Dai, Yupo Ma, Yongping Jiang

**Affiliations:** 1 Biopharmaceutical R&D Center, Chinese Academy of Medical Sciences & Peking Union Medical College, Suzhou, China; 2 Biopharmagen Corp., Suzhou, China; 3 School of Public Health, University at Albany, Albany, NY USA; 4 Department of Environmental Medicine, New York University Langone Medical Center, Tuxedo, NY USA; 5 Department of Pathology, BST-9C, The State University of New York at Stony Brook, Stony Brook, NY USA

**Keywords:** Endothelial progenitor cells, Human cord blood CD34^+^ cells, Expansion and differentiation, Hepatic sinusoidal endothelium injury, NOD/SCID mice

## Abstract

**Background:**

Transplantation of endothelial progenitor cells (EPCs)/endothelial cells (ECs) has been used for the treatment of ischemic diseases and hemophilia A, due to their great capacity for producing factor VIII and for repairing vascular damage. We established an effective approach to stimulate the expansion and differentiation of EPCs for potential therapeutic applications.

**Methods:**

CD34^+^ cells isolated from human cord blood were cultured in a two-step system for 21 days. The generated adherent cells were characterized via flow cytometry and immunofluorescent staining. Moreover, single-cell clonogenic and tube-forming assays were carried out to evaluate their potential to proliferate and form vessel networks. Furthermore, these cells were transplanted into a mouse model of hepatic sinusoidal endothelium injury by hepatic portal vein injection to investigate their in-vivo behavior.

**Results:**

The two-step culture protocol promoted the expansion and differentiation of human cord blood CD34^+^ cells efficiently, resulting in a large number of adherent cells within 3 weeks. The generated adherent cells were identified as EPCs/ECs based on the expression of CD31, CD144, vWF, and FVIII, and cell numbers showed a 1400-fold increase compared with the initial number. Moreover, these EPCs/ECs were capable of proliferating and establishing colonies as individual cells, and forming tube-like structures. More significantly, tissue examination of mice after transplantation revealed that the injected EPCs/ECs migrated and integrated into the liver, reconstituting the sinusoidal endothelial compartment.

**Conclusions:**

We developed an approach for the generation of cord blood-derived EPCs/ECs on a large scale, characterized them phenotypically, and demonstrated their in-vivo functional capacity. Our approach provides an excellent source of healthy EPCs/ECs for use in cell therapy in a clinical setting.

## Background

Endothelial differentiation was traditionally believed to occur exclusively during embryonic development after the differentiation of mesodermal cells to angioblasts [1]. In 1997, Asahara et al. [2] first proposed that CD34^+^ mononuclear blood cells isolated from human peripheral blood can differentiate ex vivo into an endothelial phenotype and participate in angiogenesis. This finding represented a milestone in the discovery and definition of endothelial progenitor cells (EPCs). Thereafter, many pioneering studies were conducted to reveal the role of EPCs in the physiological functions of wound healing and the pathogenesis of ischemic diseases [3–6]. Recently, the therapeutic applications of EPCs in treating hemophilia A (FVIII-deficient disease) were reported, because EPCs can differentiate into mature endothelial cells (ECs) to produce FVIII [7–12]. These cells can thus be regarded as an important source of FVIII, and can be used as a novel cell therapy for neovascularization.

EPCs are thought to be derived from bone marrow, and can be isolated from adult peripheral or umbilical cord blood [13–15]. However, EPCs represent a very small proportion of the cells in the circulation, ranging from 0.002 to 0.01% in peripheral blood and from 0.2 to 1% in umbilical cord blood. Although they can expand exponentially, migrate efficiently to sites of neovascularization, and differentiate into ECs in ex-vivo studies [16, 17], the number of cells is limited in the systemic infusion of allogenic EPCs in patients. Difficulties in obtaining adequate numbers of cells for transplantation currently restrict their use in clinical treatments [18].

EPCs from circulating mononuclear cells (MNCs) have been defined as early EPCs and late EPCs. Early EPCs share a number of cell-surface markers with hematopoietic stem cells (HSCs) such as CD34, CD133, and Tie-1/2 [19, 20], and secrete many stem cell growth factors such as granulocyte colony stimulating factor (G-CSF), stem cell factor (SCF), and interleukin (IL)-6/11 [21, 22]. A recent publication reported that cytokines such as SCF, FMS-like tyrosine kinase 3 ligand (Flt-3 L), and IL-3 could be applied for successful nonadhesive expansion of early EPCs for effective treatment of ischemia [23].

In the current study, an innovative and efficient culture system for the expansion and differentiation of cord blood CD34^+^ cells to generate EPCs was explored successfully. A new two-step system for EPC culture was developed, in which stem cell cytokines SCF, Flt-3 L, IL-3, thrombopoietin (TPO), and granulocyte-macrophage colony-stimulating factor (GM-CSF) combined with endothelial growth factors including vascular endothelial growth factor (VEGF), insulin-like growth factor (IGF), epidermal growth factor (EGF), fibroblast growth factor (FGF), and fibronectin (FN) were applied to the cord blood CD34^+^ cells. The generated EPCs/ECs were characterized by a specific endothelial phenotype and transplanted into nonobese diabetic/severe combined immunodeficient (NOD/SCID) mice to reconstruct the injured hepatic sinusoidal endothelium, which expressed factor VIII and other endothelial functional factors. Our study will lead to the establishment of a new expansion system for EPCs, and an improvement in the potential use of EPCs/ECs to treat ischemic diseases and hemophilia A in a clinical setting.

## Methods

### Ethics statement

All of the human cells or tissues used in this study were obtained from healthy newborn donors in Suzhou Municipal Hospital (Suzhou, China) after mothers’ written informed consent. The study was approved by the Hospital’s Ethics Committee and Research Ethics Advisory Committee (Permit Number 2014SZSLK073).

All research involving animals was conducted according to relevant national and international guidelines. NOD/SCID mice (18–22 g, male, 6–8 weeks old) were obtained from SLAC Laboratory Animal Co. (Shanghai, China), and experiments were performed in accordance with the Guidelines and Policies for Animal Surgery provided by the SPF Animal Lab (Animal Experimental Center of Soochow University, Suzhou, China). All surgeries were performed under anesthesia, and efforts were made to minimize the suffering of the animals. All experiments carried out on mice were approved by the Institutional Animal Use and Care Committee of Soochow University (IACUC Permit Number SYXK (Su) 2014-0078).

### Isolation of CD34^+^ cells from cord blood

Human umbilical cord blood samples (70–100 ml each; *n* = 10) were collected in sterile blood packs containing citrate–dextrose solution as the anticoagulant. After a Ficoll Hypaque (density 1077 g/cm^3^; GE Healthcare, Norway) density gradient centrifugation, the MNCs were isolated, washed twice, and suspended in sterile PBS with 0.5% BSA and 2 mM EDTA. CD34^+^ cells were separated from MNCs by a magnetic bead separation method (Miltenyi Biotec, Germany). In brief, the total MNCs were labeled with mouse anti human CD34 mAb coupled with microbeads (MACS). The bead-positive cells (MNC-CD34^+^) were enriched on positive-selection columns set in a magnetic field. Flow-cytometric analyses of purified cells using a PE-conjugated mouse anti human CD34 mAb (BD Biosciences, USA) showed that 94.1 ± 5.3% of the selected cells were positive for CD34.

### Cell culture of EPCs

The culture system to expand and differentiate EPCs was divided into two steps:

1. Step I (days 0–6) involved the proliferation of MNC-CD34^+^ cells. In this step, 2 × 10^5^ cells/ml were seeded into the 24-well plates in Iscove’s modified Dulbecco’s medium (IMDM) and subcultured immediately when the cell density reached over 1 × 10^6^/ml. Cytokines SCF (200 ng/ml), Flt-3 L (200 ng/ml), TPO (20 ng/ml), IL-3 (10 ng/ml), GM-CSF (12.5 ng/ml), and VEGF (50 ng/ml) were added to the IMDM medium to promote proliferation of the suspended CD34^+^ cells. The markers CD34, CD133, and VEGFR-2 (BD Biosciences, USA) were tested by flow cytometry for the identification and quantification of the CD34^+^ cells and EPCs.
2. Step II (days 7–21) involved the expansion and differentiation of EPCs from proliferated CD34^+^ cells. EBM-2 basal medium (LONZA, Switzerland) supplemented with VEGF (25 ng/ml; PeproTech, USA), IGF (20 ng/ml; PeproTech), EGF (10 ng/ml; PeproTech), b-FGF (10 ng/ml; PeproTech), FN (5 μg/ml; Sigma-Aldrich, USA), ascorbic acid (2 μg/ml; Sigma-Aldrich), heparin (100 U/ml; Sigma-Aldrich), hydrocortisone (100 ng/ml; Sigma-Aldrich), l-glutamine (4 mM; Life Technologies, USA), and 20% FBS (Life Technologies) was used to culture the EPCs. Cells were seeded at 1 × 10^6^ cells per well into the 24-well plates, which were precoated with FN (2.5 μg/cm^2^) at 37 °C for 2 h. The supernatant medium and nonadherent cells were removed on day 9, and the adherent cells were continued to be cultured in the same manner to day 21. The morphological changes were monitored using an Olympus IX51 inverted microscope (Olympus Corporation, Japan).

### Flow cytometry

Adherent cells were first detached using trypsin and collected together with suspended cells in PBS. The cells were then incubated with antibodies or the IgG isotype control for 30 min at room temperature. The antibodies used were PE-conjugated mouse anti-human CD34 mAb, APC-conjugated mouse anti-human CD133 mAb, PE-conjugated mouse anti-human VEGFR-2, APC-conjugated mouse anti-human CD31 mAb, FITC-conjugated mouse anti-human CD144 mAb, PE-conjugated mouse anti-human CD45 mAb, and PE-conjugated mouse anti-human CD14 mAb (BD Biosciences). Data were collected on a FACSVerse flow cytometer (BD Biosciences) and analyzed with FlowJo.

### Vascular tube formation assay

Prechilled 96-well plates were coated with 50 μl Matrigel basement membrane matrix (BD Biosciences) per well and incubated for 1 h at 37 °C. EPCs were seeded on top of the gelled Matrigel at 2.5 × 10^4^ cells in 100 μl step II medium. After incubating for 10 h, endothelial network formation was examined and representative fields were photographed under a microscope (Olympus Corporation).

### Immunostaining

Cells were fixed with 4% paraformaldehyde for 15 min at room temperature. Primary antibodies used were rabbit anti-human CD31 (1:500; Abcam, UK), mouse anti-human vWF (1:250; Cell Signaling Technology, USA), and mouse anti-human FVIII (1:500; Abcam). Cells then were incubated with fluorescent-labeled secondary antibodies including Cy3-labeled donkey anti-rabbit IgG (1:1000; Jackson ImmunoResearch, USA) and FITC-labeled donkey anti-mouse IgG (1:1000; Jackson ImmunoResearch). Nuclei were counterstained with 1 mg/ml 4,6-diamidino-2-phenylindole (DAPI; Sigma-Aldrich) for 10 min. The stained cells were imaged using a confocal microscope (FV1000; Olympus Corporation), and then the fluorescence intensities were measured in the resulting images using ImageJ software/Olympus.

### Western blot

Cells were washed with cold PBS and then lysed with protein extraction reagent. The lysates were centrifuged at 12,000 rpm at 4 °C for 15 min to remove cell debris and the supernatants were transferred to fresh tubes. Protein was separated using 10% SDS-PAGE gels and transferred to a 0.22 μm PVDF membrane at a constant 300 mA for 3 h using a Trans-Blot Electrophoretic Transfer Cell. After blocking with 5% BSA in TBST overnight at 4 °C, the membrane was immunoprecipitated with vWF and FVIII primary antibody (rabbit mAb anti-vWF, 1:1000 and mouse mAb anti-FVIII, 1:500; Abcam) for 1.5 h at room temperature. Membranes were then washed and incubated for 1 h at room temperature with the appropriate horseradish peroxidase-linked secondary antibody (1:1000; Abcam).

### Enzyme-linked immunosorbent assay

Culture supernatants were filtered through a 0.22-μm filter (Millipore, USA) and subjected to 10-fold concentration. The concentrates of secreted FVIII proteins were tested by a commercial enzyme-linked immunosorbent assay (ELISA) kit according to the manufacturer’s protocol (Sekisui Diagnostics, USA).

### Single-cell clonogenic assay

EPCs were serially diluted and plated onto a 96-well plate. The wells were examined under an Olympus IX51 inverted microscope (Olympus Corporation) and only wells containing a single cell were monitored. After 1 week of culture, wells were examined for colony growth by visual inspection.

### Preparation of GFP lentivirus vector and infection of EPCs/ECs

The green fluorescent protein (GFP) lentivirus vector (pSSI13772) was prepared as described previously [12, 24]. Before transplantation, the differentiated/expanded EPCs/ECs were infected with GFP lentivirus for 2 h supplemented with polybrene at 8 μg/ml. GFP expression was observed using a fluorescence microscope (Olympus Corporation). Over 90% of EPCs/ECs showed green fluorescence 24 h post infection. Trypsin was used to detach the cells, which were then centrifuged and suspended in sterile PBS.

### Transplantation of EPCs/ECs into NOD/SCID mice

NOD/SCID mice were housed in individual stainless steel cages in a SPF facility in Soochow University, with a regulated temperature of 24 ± 2 °C, relative humidity of 50 ± 10%, and a 12-h light cycle. Forty NOD/SCID mice were randomly divided into five groups, namely control group, blank group, trans-7d group, trans-14d group, and trans-28d group. Except for the blank group, acute liver injury was induced in mice by an intraperitoneal injection of 100 mg/kg monocrotaline (MCT; Sigma-Aldrich) in saline 24 h prior to the cell transplantation [25]. After anesthesia with FFM mix (2.5 mg/kg fluanisone, 0.105 mg/kg fentanylcitrate, and 1.25 mg/kg midozalam HCl in H_2_O), mice in the blank, trans-7d, trans-14d, and trans-28d groups were transplanted with EPCs/ECs (6 × 10^6^ in 100 μl PBS per mouse) via the hepatic portal vein using 27-gauge needles (Hamilton, Switzerland) [12], while the control group mice were treated with 100 μl PBS. At days 7, 14, and 28 after transplantation, the mice in the trans-7d, trans-14d, and trans-28d groups were sacrificed by cervical dislocation, respectively, and the livers were removed and fixed in 4% formalin for analysis. In control and blank groups, mice were sacrificed and livers were analyzed 7 days after transplantation.

### Detection of transplanted EPCs/ECs

The hepatic tissues were embedded in Optimum Cutting Temperature compound (OCT; Sakura, Tokyo, Japan) to make the cryosections (20 μm; Leica CM1900, Germany). Twenty-five cryosections were randomly selected from the liver pieces of each mouse. Immunofluorescent staining was used to identify EPCs/ECs infected with the GFP lentivirus. Cryosections were labeled with rabbit anti-GFP primary antibody (1:100; Santa Cruz, USA) and Cy3-labeled donkey anti-rabbit IgG secondary antibody (1:1000; Jackson ImmunoResearch). Nuclei were counterstained with 1 mg/ml DAPI (Sigma-Aldrich) for 10 min. These cryosections were then observed under a fluorescence microscope (Olympus Corporation). The percentages of GFP staining cells were quantified using ImageJ software.

### Statistical analysis

Except for the in-vivo mouse experiment, which was repeated twice, all other experiments were repeated at least three times. All results are expressed as mean ± SD. All data were subjected to ANOVA followed by Fisher’s analysis for comparison between two means.

## Results

### Proliferation of cord blood CD34^+^ monocytes in step I of culture

**Fig. 1:**
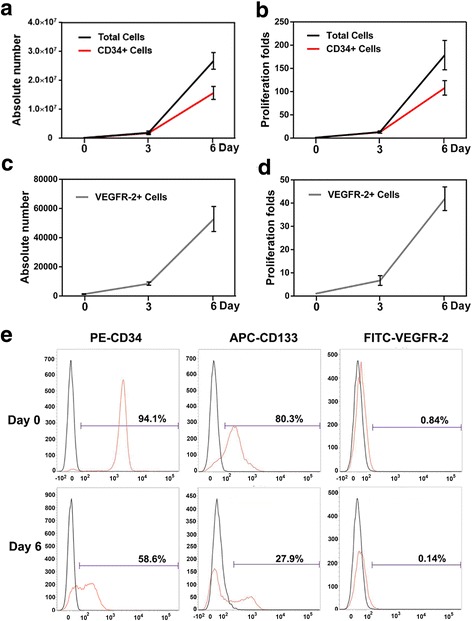
Proliferation of CD34^+^ monocytes and early EPCs derived from human cord blood. The cord blood CD34^+^ cells were cultured for 6 days in the modified IMDM medium supplemented with a cytokine cocktail. **a** Absolute number of total cells and CD34^+^ cells from day 0 to day 6. **b** Fold-increase in proliferation of total cells and CD34^+^ cells from day 0 to day 6. **c, d** Absolute number and fold-increase of VEGFR2^+^ cells from day 0 to day 6. **e** Representative flow cytometry profiles of CD34, CD133, and VEGFR2 markers on cells before and after expansion. Data are mean ± SD; *n* = 10

**Fig. 2:**
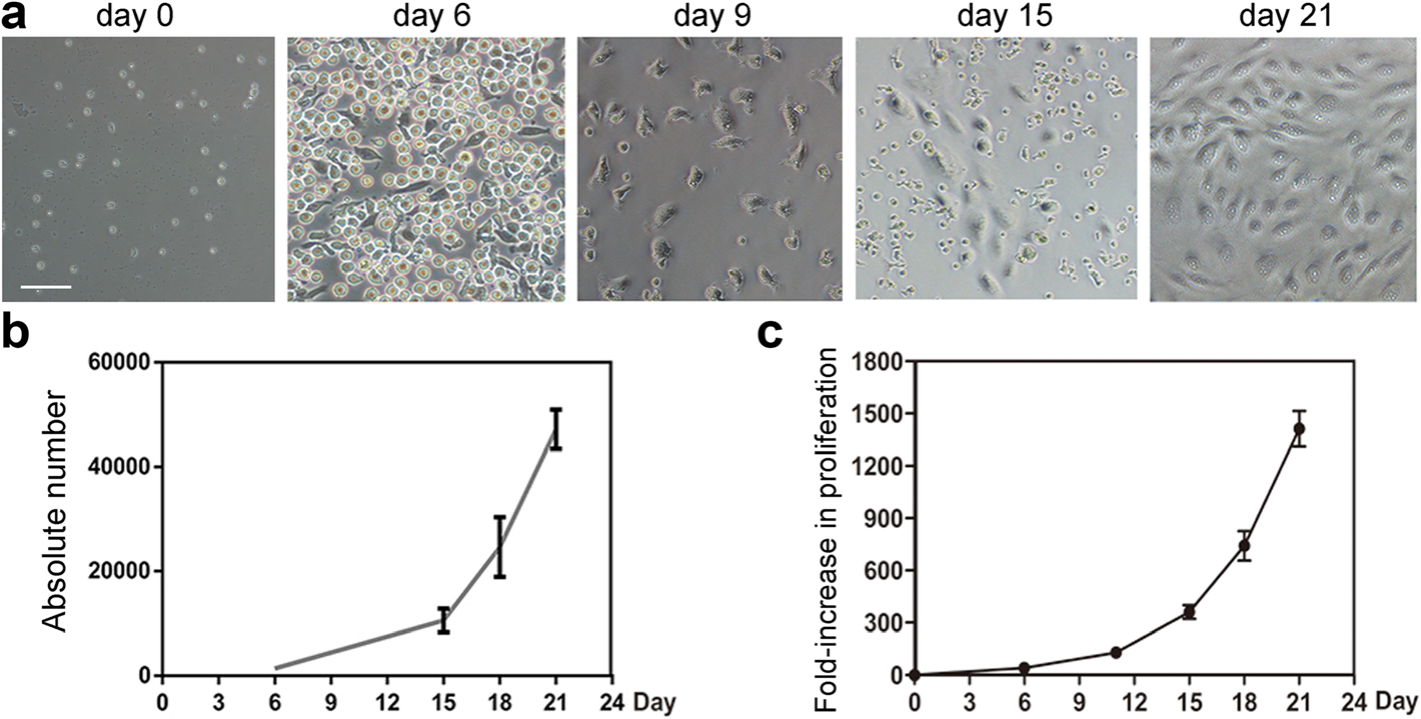
Expansion and differentiation of EPCs from proliferated CD34^+^ cells. The ex-vivo expanded human CD34^+^ cells were cultured with the optimized medium recipe to induce EC differentiation (*scale bar* = 50 μm). **a** Cell morphology imaged with an optical microscope on days 0, 6, 9, 15, and 21. **b** Absolute number of VEGFR2^+^ cells at different time points. **c** Fold-increase in proliferation of VEGFR2^+^ cells at different time points. Data are mean ± SD; *n* = 10

The number of CD34^+^ cells has been reported to correlate positively with the endothelial differentiation rate [23]. In the first step of our culture protocol, a cytokine combination including SCF, Flt-3 L, TPO, IL-3, GM-CSF, and VEGF was used to efficiently expand cord blood CD34^+^ cells, as well as early EPCs. After 6 days in culture, the total cell number increased from 1.5 × 10^5^ to 2.68 × 10^7^ ± 2.90 × 10^6^ cells, showing a ~180-fold proliferation (Fig. 1a, b). The expression of CD34, CD133, and VEGFR2 markers on days 0 and 6 is shown in Fig. 1e. The initial percentage of CD34^+^, CD133^+^, and VEGFR2^+^ cells was 94.1 ± 5.3%, 80.3 ± 2.7%, and 0.84 ± 0.16%, respectively. On day 6, CD34^+^ cells were maintained at the relatively high level of 58.6 ± 2.7%, and CD133^+^ and VEGFR2^+^ cells decreased to 27.9 ± 1.8% and 0.14 ± 0.02%, respectively. Within 6 days, the fold-increase of CD34^+^ and VEGFR2^+^ cells was 108 ± 15.6 and 41.9 ± 5.1, respectively (Fig. 1b–d). Meanwhile, cells exhibited robust suspension growth, with only a small number of cells showing an adhesive phenotype as early EPCs (Fig. 2a, day 6). These results indicated that CD34^+^ cells and early EPCs both undergo extensive proliferation in step I of the culture protocol.

### Expansion and differentiation of EPCs in step II of culture

Six days after the initial expansion of step I, cells were transferred to EBM-2 basal medium supplemented with endothelial growth factors VEGF, IGF, EGF, FGF, and FN. At various time points, morphological changes in cultured cells could be observed clearly with a light microscope (Fig. 2a). Three days later (day 9), the suspended early EPCs became adherent to the plates, and started to grow exponentially. From day 15, a typical spindle-like shape could be observed clearly in the cultured cells. After 21 days following this culture protocol, the differentiated/expanded EPCs/ECs were collected for analysis. By day 21, the absolute number of VEGFR2^+^ cells reached 4.72 × 10^4^ ± 3.74 × 10^3^ cells per well, from 1.4 × 10^3^ cells per well on day 6, meaning that the EPCs/ECs ultimately underwent a 1412.03 ± 102.25-fold increase over the initial numbers of EPCs (Fig. 2b, c). Collectively, these results demonstrated that this innovative two-step culture system was effective for the ex-vivo expansion and differentiation of EPCs/ECs derived from cord blood CD34^+^ cells.

### Characterization of differentiated EPCs/ECs

**Fig. 3:**
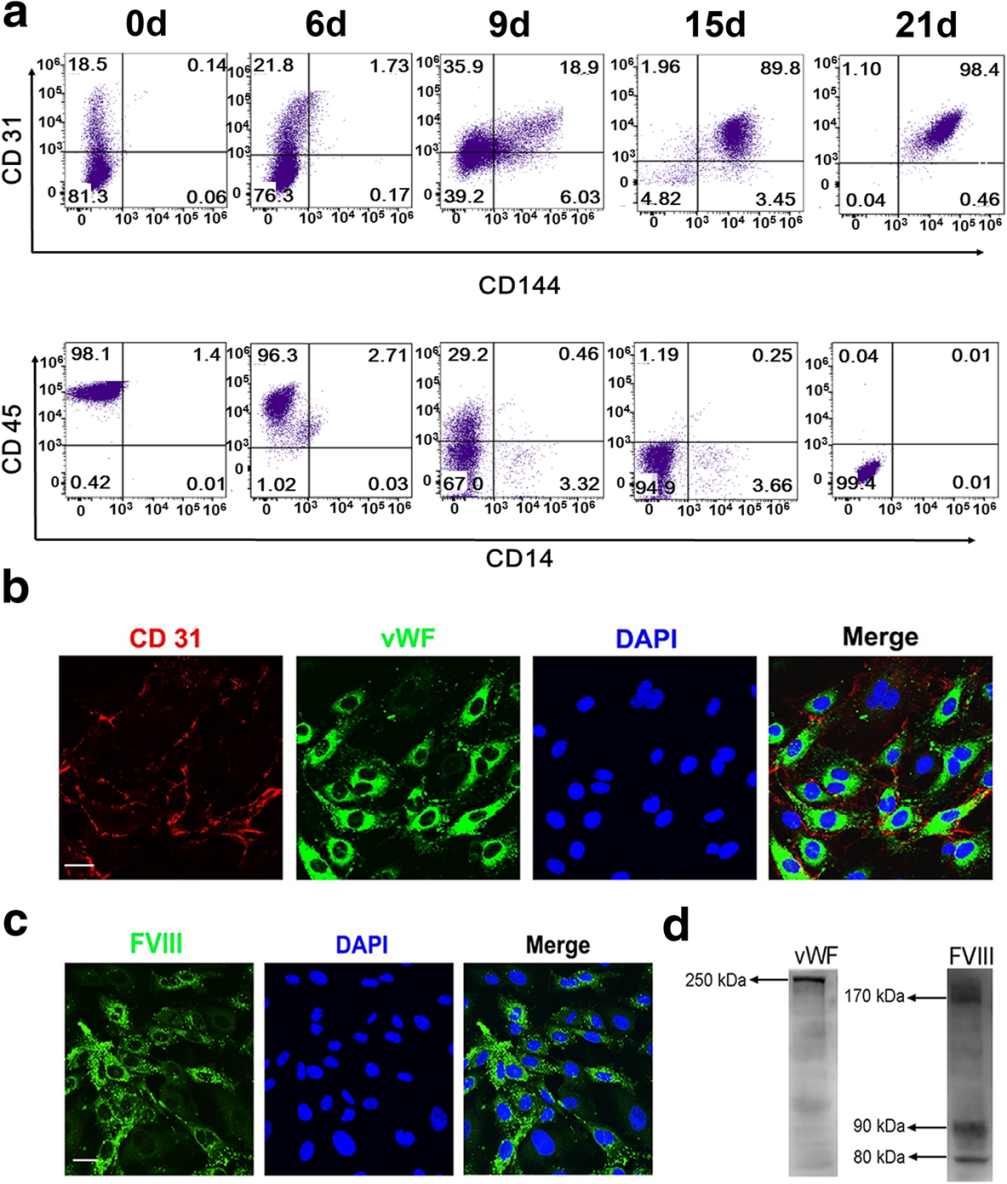
Phenotypic and functional analysis of differentiated EPCs/ECs. **a** Representative flow cytometry profiles of CD31, CD144, CD45, and CD14 cell markers on days 0, 6, 9, 15, and 21. **b** Immunofluorescence analysis of CD31 (*red*) and vWF (*green*) double staining on day 21 (*scale bar* = 20 μm). **c** Immunofluorescence staining of FVIII in cytoplasm on day 21 (*scale bar* = 20 μm). **d** Western blot analysis of cytoplasm FVIII and vWF proteins on day 21 (Color figure online)

The cell surface markers during the cell culture process were monitored by flow cytometry on days 0, 6, 9, 15, and 21. During the differentiation, the expression levels of EC specific markers CD31 and CD144 were increasing continuously, with the frequency of CD31^+^/CD144^+^ late EPCs/ECs achieving 99.0 ± 0.8% by day 21 (Fig. 3a, upper panel). Moreover, the percentage of CD45^+^ and CD14^+^ cells was 0.04 ± 0.01% and 0.02 ± 0.01%, respectively, in week 3, indicating a very small amount of white blood cells and macrophages in the cell preparations (Fig. 3a, lower panel). Immunofluorescence analysis of differentiated cells showed that over 90% of the adherent cells expressed CD31 and vWF by double staining with Cy3 and FITC-conjugated antibodies (Fig. 3b), consistent with the results obtained by flow cytometric analysis. Moreover, immunofluorescent staining indicated that over 80% of adherent cells were positive for FVIII, indicating the successful expression of FVIII in the cell cytoplasm (Fig. 3c). Meanwhile, the expressions of cytoplasm FVIII and vWF proteins were confirmed by western blot analyses (Fig. 3d). In addition, more than 20 ng/ml of FVIII secreted in the culture media was detected on day 21 to further confirm the FVIII secreting capacity of induced EPCs/ECs.

**Fig. 4:**
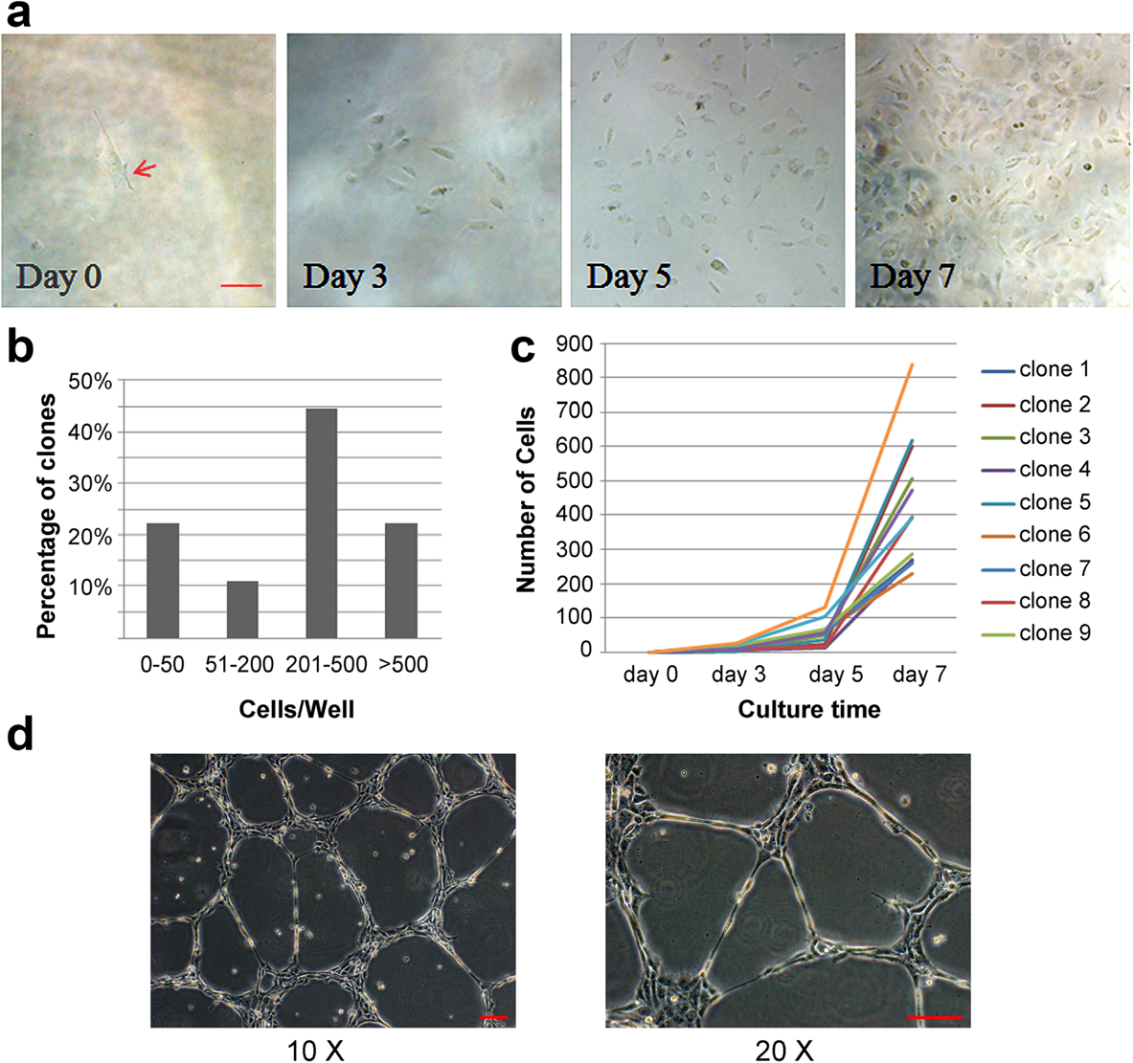
Clonogenic quantitation of single EPC and vessel-like structures of differentiated ECs. **a** Cell morphology at different time points by seeding a single differentiated EPC in an individual well of a 96-well plate. The arrow indicates a single colony on day 0. **b, c** Percentage and number of cell progeny at different time points. **d** Differentiated EPCs/ECs were placed on Matrigel for a 10-h incubation, and representative fields of view showing endothelial networks were photographed under a microscope (*scale bars* = 100 μm)

In addition, the clonogenic and proliferative potential of differentiated EPCs was investigated by a single-cell assay to determine whether a single EPC could proliferate and form a colony in the absence of other cells. As shown in Fig. 4a, the single plated EPC showed cell division from day 3, and formed obvious colonies on day 5. From day 5 to day 7, the small colonies formed showed robust proliferation, and the average number of progeny derived from a single plated cell was more than 350 cells on day 7. About 20% of the single EPCs gave rise to large colonies or clusters containing over 500 cells (Fig. 4b, c). Furthermore, the tube formation assays on Matrigel showed that cells arranged themselves into capillaries and formed vascular networks with branching points (Fig. 4d).

### Transplantation of cultured human EPCs/ECs into the livers of NOD/SCID mice

**Fig. 5:**
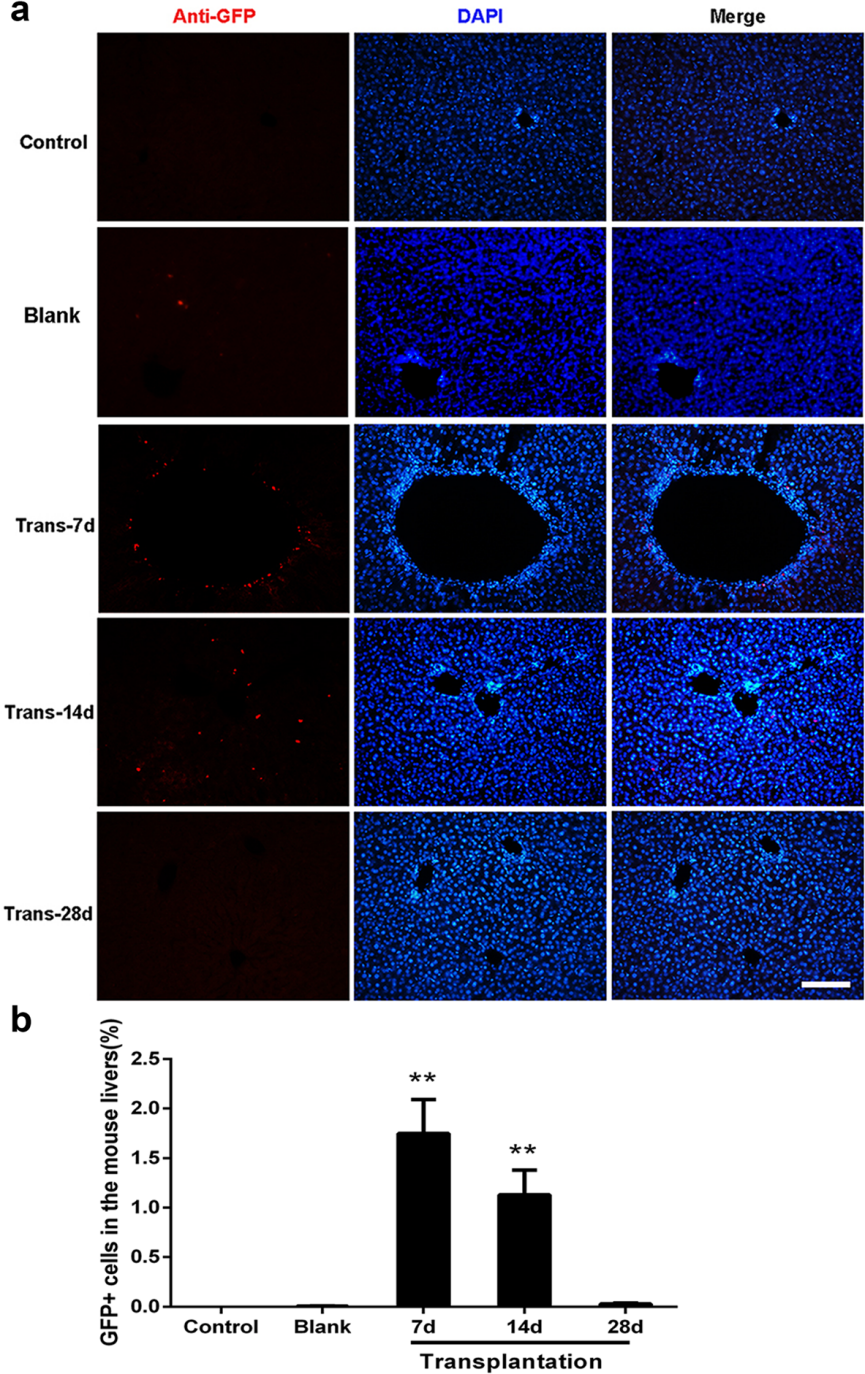
Transplantation of human EPCs/ECs into the livers of NOD/SCID mice. **a** Acute liver injury was induced in mice by an intraperitoneal injection of 100 mg/kg MCT in the control, trans-7d, trans-14d, and trans-28d groups. EPCs/ECs infected with GFP lentivirus were transplanted into mice in the blank, trans-7d, trans-14d, and trans-28d groups (6 × 10^6^ cells in 100 μl PBS per mouse) via the hepatic portal vein while the control group mice were treated with 100 μl PBS. At days 7, 14, and 28 after transplantation, mice in the trans-7d, trans-14d, and trans-28d groups were sacrificed by cervical dislocation, respectively, hepatic tissues were obtained to make cryosections (20 μm), and the GFP-positive cells were detected using immunofluorescent staining. In the control and blank groups, mice were sacrificed and hepatic tissues were analyzed 7 days after transplantation. *Scale bar* = 50 μm. **b** Percentages of GFP-positive cells in the mouse livers were analyzed by ImageJ software. Twenty-five cryosections per mouse (*n* = 8 each group) were calculated. Data are mean ± SD. **P < 0.01. *DAPI* 4,6-diamidino-2-phenylindole, *GFP* green fluorescent protein

In order to determine the efficacy of the produced EPCs/ECs in vivo, an acute hepatic sinusoidal endothelium injury model induced by MCT was established in NOD/SCID mice. The EPCs/ECs infected with a GFP lentiviral vector were injected into mice, and at indicated time points post transplantation the livers were obtained and analyzed by immunostaining with anti-GFP primary antibody and Cy3-labeled secondary antibody. The immunostaining results revealed that there were almost no GFP-positive cells in the control and blank groups, but these GFP-positive cells obviously existed in the hepatic sections in trans-7d and trans-14d groups (Fig. 5a). The transplanted cells were observed mostly around the endothelium of small veins in the hepatic issues on day 7, while they were found scattered in the intercellular spaces between hepatocytes at the hepatic plates on day 14. Furthermore, quantitative analysis showed that about 1.75 ± 0.34% and 1.13 ± 0.25% of liver cells were observed as GFP-positive staining cells in the livers on days 7 and 14, respectively (Fig. 5b). These results indicated the cultured EPCs/ECs could migrate into the injured sites and structurally reconstitute the sinusoidal–endothelial compartment in the liver.

## Discussion

EPCs are an attractive resource for enhancing neovascularization in ischemic tissue and producing FVIII in cases of hemophilia A. However, the low number of cells obtained after purification from peripheral or cord blood is a common barrier to the clinical use of EPCs. Here, we developed an innovative two-step system for high-level ex-vivo expansion and differentiation of human EPCs derived from cord blood CD34^+^ cells. Using this protocol, human EPCs/ECs were efficiently generated from human cord blood CD34^+^ cells within 3 weeks, achieving a more than 1400-fold increase in proliferation. Compared with the approximately 400-fold increase reported in previous studies [17, 26], our technique showed a remarkable improvement in the large-scale generation of EPCs/ECs ex vivo. More than 2 × 10^8^ EPCs/ECs were generated from one cord blood sample (70–100 ml) within 3 weeks, and afterwards these EPCs/ECs could also be sustainably cultured ex vivo for up to 3 months, accompanied by a process of gradual maturation [27]. This production platform could therefore provide sufficient functional EPCs/ECs for transplantation treatments in a clinical setting.

In our study, human umbilical cord blood was use used to derive CD34^+^ cells for EPC culture. In contrast to progenitor cells derived from adult bone marrow and adult peripheral blood, cord blood stem cells possess distinct proliferation advantages in cell-cycle rate and telomere length [23]. Moreover, obtaining cord blood is noninvasive and the cells are genomically stable, in contrast with the invasive procedure of bone marrow isolation to harvest induced pluripotent cells (iPSCs) [28]. In addition, all of the cytokines included in the culture system, such as SCF, TPO, FLT-3, IL-3, GM-CSF, and VEGF, were biological endogenous factors and are commonly used in clinical treatments. This treatment would pose no risk of cell toxicity or tumor stimulation. The convenience of sample collection and safety of an ex-vivo culture system could be easily adapted for large-scale production of EPCs/ECs for clinical applications.

In contrast to previous methods, in step I of culture (days 0–6) we neither induced adherence in the EPC culture from the beginning nor simply expanded the CD34^+^ cells in suspension; we used a combination of high-level VEGF (50 ng/ml) with other stem cell growth factors (SCF, TPO, FLT-3, IL-3, and GM-CSF). Under the stimulation of this novel cytokine combination, CD34^+^ cells, including the early EPCs, maintained a robust proliferation rate without significant changes in morphology. Within 6 days, the proliferation fold of CD34^+^ cells and VEGFR-2^+^ cells was 108 ± 15.6 and 41.9 ± 5.1, respectively, which determined the final yield of expanded EPCs.

In step II of culture (days 7–21) total cells were transferred to the EBM-2 medium in FN-coated plates, and a low concentration of VEGF (25 ng/ml) combined with other endothelial growth factors (IGF, EGF, and b-FGF) was added to enhance the generation of EPCs. This culture condition on the one hand promoted the proliferated CD34^+^ cells to further differentiate towards endothelial lineage, and on the other stimulated the existing and newly generated EPCs to adhere to the plates and grow exponentially. On day 21, a large number of functional EPCs/ECs were obtained with a purity of 99% (Fig. 3).

As regards the efficiency evaluation of EPCs/ECs, we developed a NOD/SCID mouse model with hepatic sinusoidal endothelium injury to verify the engraftment capacity of generated human EPCs/ECs [7]. Because of the high cross-reactivity of human and mouse functional proteins, we were unable to detect human FVIII or vWF in the mouse liver sections. However, we verified the endothelial secretion of these proteins before transplantation (Fig. 3b, c). Moreover, in order to enhance the fluorescence signal in GFP-positive cells, we used an anti-GFP primary antibody followed by a fluorescently labeled secondary antibody to verify the presence of human EPCs/ECs. The results from day 7 post transplantation showed that most of the GFP-positive cells could be observed around the endothelium of small veins in the hepatic issues. Subsequently, on day 14, they were found to scatter in the intercellular spaces between hepatocytes at the hepatic plates. These results indicated that the human EPCs/ECs first adhered to the vascular inner wall after injection via the hepatic portal vein, and then gradually migrated to the sites of injury induced by MCT in the hepatic sinusoidal endothelium. These observations revealed significant chemotaxis toward the injured sites and migration activity of the injected human EPCs/ECs. However, no GFP-positive cells were detected on day 28. One possible explanation for this is that GFP is strongly immunogenic [29, 30], so implanted EPCs/ECs expressing GFP might be ablated gradually by the host due to immunological rejection. In addition, the current mouse model was established with NOD/SCID mice, which can have activated macrophages that may cause destruction of donor cells.

During a 6-month observation period, besides the MCT-induced injury, no tumor formation or other induced pathological problems were detected in our study. On the basis of the in-vivo studies, we can claim that the human EPCs/ECs produced using our two-step system can successfully survive and safely exist in vivo for more than 2 weeks. If this approach is applied to the treatment of hemophilia A, the persistent FVIII secretion will innovatively reduce patients’ dependency on expensive recombinant human FVIII. In addition, because human leukocyte antigen (HLA) matching is extremely strict for ECs, transplantation of autologous EPCs/ECs as a personalized treatment may also provide satisfactory therapeutic effects in clinical applications. We also achieved successful expansion and differentiation of EPCs derived from human mobilized peripheral blood CD34^+^ cells by the two-step system, further improving the clinical value of this technology.

## Conclusion

We describe an approach for the generation of cord blood-derived EPCs/ECs on a large scale, characterize them phenotypically, and demonstrate their in-vivo functional capacity. Our study strongly indicates that the two-step culture system can be developed into an innovative technology for industrial production of human EPCs/ECs derived from cord blood. In our future work, we will further evaluate the safety and efficacy of this technology in a nonhuman primate model. This preclinical research will promote the cell therapy application of high-efficiency ex-vivo expansion of EPCs in ischemic disease and hemophilia A.

## Abbreviations

FITC: Fluorescein isothiocyanate
Flt-3 L: Fms-related tyrosine kinase 3 ligand
GFP: Green fluorescent protein
HSC: Hematopoietic stem cell
MACS: Magnetic-activated cell sorting
MNC: Mononuclear cell
NOD/SCID: Nonobese diabetic/severe combined immunodeficiency
PBS: Phosphate-buffered saline
PE: Phycoerythrin
SCF: Stem cell factor
SRC: SCID repopulating cells
TPO: Thrombopoietin
